# A Review of Energy Drinks and Mental Health, with a Focus on Stress, Anxiety, and Depression

**DOI:** 10.1089/jcr.2015.0033

**Published:** 2016-06-01

**Authors:** Gareth Richards, Andrew P. Smith

**Affiliations:** Centre for Occupational & Health Psychology, School of Psychology, Cardiff University, Cardiff, United Kingdom.

## Abstract

***Background:*** Concerns have been expressed regarding the potential for caffeinated energy drinks to negatively affect mental health, and particularly so in young consumers at whom they are often targeted. The products are frequently marketed with declarations of increasing mental and physical energy, providing a short-term boost to mood and performance. Although a certain amount of evidence has accumulated to substantiate some of these claims, the chronic effects of energy drinks on mental health also need to be addressed.

***Methods:*** To review the relevant literature, PubMed and PsycINFO were searched for all peer-reviewed articles published in English that addressed associations between energy drink use and mental health outcomes. Case reports were also considered, though empirical studies investigating acute mood effects were excluded as a review of such articles had recently been published. Fifty-six articles were retrieved: 20 of these (along with eight more identified through other means) were included in the current review, and, because the majority addressed aspects of stress, anxiety, and depression, particular focus was placed on these outcomes.

***Results:*** Though a number of null findings (and one negative relationship) were observed, the majority of studies examined reported positive associations between energy drink consumption and symptoms of mental health problems.

***Conclusions:*** Though the findings imply that energy drink use may increase the risk of undesirable mental health outcomes, the majority of research examined utilized cross-sectional designs. In most cases, it was therefore not possible to determine causation or direction of effect. For this reason, longitudinal and intervention studies are required to increase our understanding of the nature of the relationships observed.

## Introduction

Energy drinks (sometimes referred to as “stimulant drinks”^1^) are caffeinated soft drinks that claim to boost performance and endurance.^2^ They should not be confused with sports drinks, which are instead marketed to rehydrate and replace electrolytes lost through exercise.^3^ Although highly caffeinated soft drinks appeared in Europe and Asia in the 1960s,^4^ energy drinks as we know them initially became available to the public with the introduction of Red Bull^®^ to Austria in 1987, and subsequently to North America in 1997. The energy drinks industry has since grown exponentially, becoming a multibillion dollar market.^5^ Considering that the consumption of such products has been associated with a number of very serious health complaints,^6^ it is considered necessary to develop a better understanding of their effects in the interests of public health.

Concern has been expressed due to the caffeine content of energy drinks (reportedly sometimes as high as 505 mg per serving^4^) having the potential to cause intoxication.^6^ In addition, such products often contain numerous other substances, such as taurine, l-carnitine, glucuronolactone, B-vitamins, ginseng, and guaraná. Some additives (e.g., guaraná, kola nut, yerba maté, cocoa) may also increase overall caffeine content unbeknownst to the consumer, because in some countries, manufacturers are not required to list that which is attributable to herbal supplements in the nutritional information.^6^ It is also concerning that these additional substances are often under-studied and unregulated, and that interactions between them (as well as potentially with prescription drugs) are not yet fully understood.^6^ Furthermore, as these additives vary considerably both in presence and in concentration between products, a general problem with research in the area is that it can be difficult to compare like for like.

### Caffeine and mental health

It is important to consider relationships between mental health and caffeine use, as the substance appears to be the main active ingredient in energy drinks.^7^

Although caffeine consumption is moderately associated with a number of psychiatric disorders, the relationships appear not to be causal,^8^ and discrepancies in the literature are common.^9^ Some studies have observed positive effects: for example, low doses have been shown to elevate mood.^10^ Evidence suggests, however, that such outcomes likely depend on the dosage consumed; Kaplan *et al.*,^11^ for instance, reported that 250 mg increased elation, whereas 500 mg increased irritability. Acute effects may also vary between studies depending on whether or not the research participants in question were tested in a state of caffeine withdrawal. Further to this, baseline characteristics of caffeine consumers are likely to differ from nonconsumers. For instance, when investigating daily caffeine consumption in psychology students, Gilliland and Andress^12^ reported that trait anxiety and depression were higher in moderate and high consumers compared to abstainers.

Considering the idea that energy drink use may cause behavioral problems^13^ and negatively impact on mental health and well-being, it is concerning to find that the products are often aggressively marketed at young people.^4^ Children aged between 12 and 17 are among the fastest growing population of caffeine users,^14^ with 30–50% of adolescents and young adults being known to consume energy drinks.^6^ However, as black markets in junk food are known to exist in secondary schools, and that they may be a result of restrictive policies intended to improve the diets of children,^15^ banning the sale of energy drinks to minors could be an overly simplistic and ineffective solution.

Although the media often portrays energy drinks in a negative light, Suzuki^16^ reported that nutritional and tonic drinks (under which label they can be classified^17^) comprised 43.1% of complementary and alternative medicines used in Japan. Smith and Atroch^18^ also noted that drinks containing guaraná have been used for medicinal purposes in Brazil for hundreds of years. Due to such conflicting accounts, it is important to consider whether energy drinks do indeed affect mental health, and if so, are the effects positive, negative, or variable.

### Acute effects of energy drink consumption on mood

Energy drink companies often market their products with claims of boosting physiological functioning, providing short-term boosts to mood and performance. A current review article,^17^ as well as several more recently published reports,^19–21^ suggests that there may be some efficacy to these claims. For instance, double-blind trials have shown benefits of energy drinks compared to placebo in relation to well-being, vitality, and social extrovertedness,^22^ depression and anxiety,^21^ and in improving or maintaining mood under fatiguing or cognitively demanding tasks.^23^ However, Scholey and Kennedy^24^ observed no mood effects in relation to drinks containing caffeine, glucose, ginseng, and ginkgo biloba. A recent study by Grasser *et al.*^25^ also reported no difference in perceived stress between energy drinks and water conditions after a stress-inducing mental arithmetic task.

Negative effects have also sometimes been reported. For instance, Wesnes *et al.*^21^ observed that tension/anxiety scores (measured using the Profile of Mood States) increased significantly in the energy drink condition relative to placebo at 1 h postconsumption (although no such effect was detected thereafter). Salinero *et al.*^19^ also observed increased nervousness, insomnia, and activeness when energy drinks were consumed rather than placebos; each of these effects occurred in females, but only the effect of insomnia was statistically significant in males. However, due to the placebo condition also containing the same ingredients other than caffeine (i.e., water, taurine, sodium bicarbonate, l-carnitine, and maltodextrin), these effects may be attributable to caffeine rather than to energy drinks *per se*.

Although beneficial acute mood effects of energy drinks have been frequently reported in the literature, null findings and undesirable side effects have also been observed. Furthermore, manufacturers have rarely addressed the potential long-term effects that consuming the products may have. For this reason, the current article aims to review the literature relating to chronic energy drink use and its associations with mental health outcomes.

## Method

**FIG. 1.**
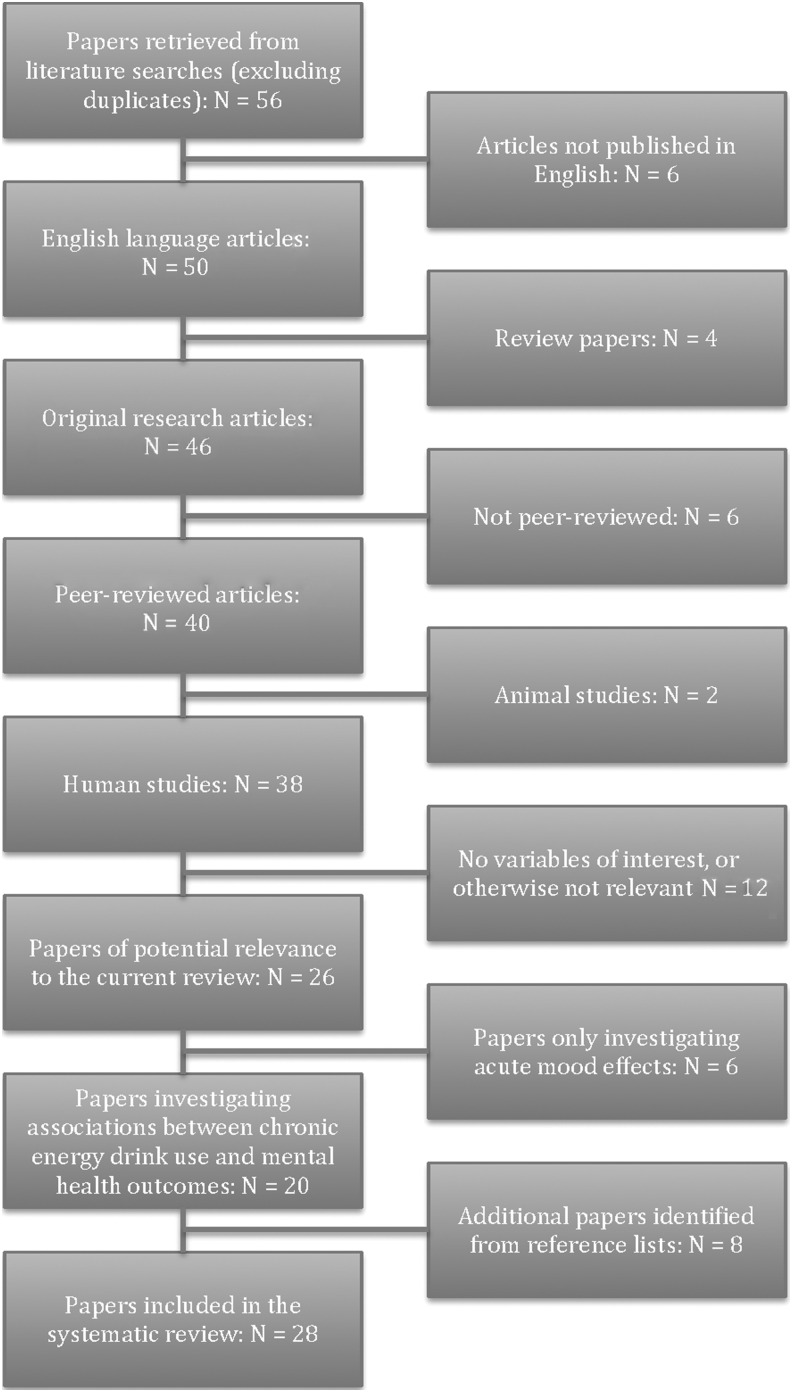
Flowchart showing the inclusion/exclusion of studies used in the systematic review of energy drinks and mental health.

PubMed and PsycINFO were searched for English language articles published between 1990 and 2015, and the following search terms were used: “energy drinks” and “mental health,” “well-being,” “wellbeing,” “stress,” “anxiety,” “depression.” Excluding duplicates, 56 articles were initially identified (for a flow diagram of their inclusion/exclusion, Fig. 1). Author GR read each of the abstracts and acquired and read all articles deemed potentially relevant. Of these, 20 (along with eight others identified by reading through reference lists) were included in the review, with the findings of case reports being considered separately to those of empirical studies.

## Results

### Case reports of energy drink consumption and mental health problems

Initial anecdotal evidence to suggest that energy drink use can be associated with the occurrence/reoccurrence of psychiatric symptoms comes from eight articles presenting case reports (Table 1). Of these, only three were identified by the initial literature search; the other five were identified from references made in other articles. Although some of the case reports relate to phenomena outside the general scope of the current review, they are included because they provide a useful starting point from which to examine associations between energy drink use and mental health. Together, 12 cases have been described, and links have been made between the use of energy drinks and the exacerbation/relapse of symptoms in patients with bipolar disorder,^26–28^ Cluster B personality disorder,^28^ and schizophrenia.^28–30^ Although these cases imply that the excessive consumption of energy drinks may act as a trigger for relapse in certain vulnerable people with pre-existing mental health problems, cases have also emerged in which serious psychiatric symptoms have occurred in otherwise healthy individuals. These reports have linked excessive energy drink consumption to anxiety,^31^ severe manic symptoms,^32^ and acute suicidality.^33^ However, although the individuals in question described by Sharma^32^ and Szpak and Allen^33^ had no previous psychiatric issues, family histories of mental health problems were revealed. The only case in which no such prior susceptibility emerged was that presented by Berigan.^31^ However, given the symptomatology and extreme consumption (6–8 8oz cans per day for 4 months), this case may simply reflect the effects of caffeine toxicity. This idea is supported by findings from other case studies, which have reported high caffeine consumption to be capable of inducing manic symptoms.^34,35^

**Table 1.** Published Case Reports Relating Psychiatric Symptoms to Energy Drink Consumption

	*Case details*	*Psychiatric history*	*Energy drink use*	*Presentation symptoms*
Berigan^31^	25-year-old male	No prior diagnosis, no chronic medical issues, or family history of psychiatric problems	6–8 (8oz) cans daily for previous 4 months	Anxiety, restlessness, fidgetiness, irritability, difficulties concentrating, problems falling asleep
Cerimele *et al*.^29^	43-year-old male	Schizophrenia (Paranoid Type), and alcohol dependence (in full sustained remission)	Began use 8 weeks before hospitalization; use escalated to 8–10 (16oz) cans daily	Paranoia, religious delusions, agitation
Chelben *et al.*^28^	Case 1: 41-year-old female	Long history of psychiatric disorder; primarily Cluster B personality disorder with salient hysterical attributes, a tendency toward dramatization, impulsivity, and suicide attempts in response to relatively low-scale triggers	At least five a day (considerably more on some days) for 1 week; consumption stopped immediately before hospitalization due to running out of money	Severe psychomotor agitation, hypervigilance, verbal and physical aggression, impulsive behavior, low threshold for aggressive outbursts
	Case 2: 38-year-old female	Comorbid bipolar disorder and borderline personality disorder, and a long history of multiple substance abuse	5–10 energy drinks per day for 1 month	Moderate psychomotor agitation, increased alertness, insomnia impulsivity, self-mutilation ideation
	Case 3: 25-year-old male	Schizophrenia	8–9 cans of energy drink at a time for once month	Psychomotor unease, hypervigilance, verbal aggression, intensive preoccupation with thoughts of death
Machado-Vieira, *et al.*^26^	36-year-old white male	Bipolar I (DSM-IV)	1 week before episode drank three cans of Red Bull at night; 3 days later drank three more cans	Mania: euphoria, hyperactivity, insomnia, increased libido, irritability
Menkes^30^	27-year-old New Zealand Maori male	Schizophrenia; previously used alcohol and cannabis to excess; currently drank up to 10 cups of instant coffee per day	First incident: two Demon Shots an hour apart; Second incident: three Demon Shots in 15 min	First incident: unease, irritability, paranoia; Second incident: restlessness, withdrawal, argumentativeness, rapid pulse, insomnia
Rizkallah *et al.*^27^	Case 1: 40-year-old male	Bipolar Type I (DSM-IV), prior intranasal cocaine dependence	Up to six small cans a day for 1 week	Manic episode: elated mood, irritability, grandiosity
	Case 2: 30-year-old female	Bipolar Type II (DSM-IV), intranasal cocaine dependence	Several incidents of using up to eight small cans a day during previous month; this pattern occurred every day for 2 weeks before admission	Irritability, flight of ideas, reduced need for sleep, heightened sexually oriented activities
	Case 3: 36-year-old male	Bipolar Type I (DSM-IV), cannabis dependence and cocaine abuse	Up to nine small cans almost daily for 2 weeks	Sleep disturbance, increased daytime sleepiness, irritability, anxiety, and depression
Sharma^32^	32-year-old German male	No prior diagnoses, no psychiatric history (other than occasional mood swings). Family history of mental illness (postpartum depression and suicide)	Began drinking Red Bull 4 weeks before admission; one to two cans daily escalated to six to eight large (550 mL) cans daily during a week before hospitalization	Decreased sleep requirement, hyperactivity, pressured speech, racing thoughts, delusions of grandiosity and paranoia, risk-taking behavior, and lack of insight
Szpak and Allen^33^	28-year-old male professional boxer	No personal history of psychiatric problems, although one brother committed suicide, another died from a drug and alcohol overdose (unclear if intentional or not), and his father became an alcoholic	Drank 14 (250 mL) cans of energy drink in the day and evening (7 each consecutive day)	Acute suicidality following sleep deprivation

Although the case reports presented in this section cannot prove that a causal relationship exists between energy drink use and the acute onset of psychiatric problems, the chronicity of such accounts is compelling. These reports are also not necessarily indicative of energy drinks being a problem when used moderately by the general population. To address this concern, the next section will present findings from studies investigating chronic energy drink consumption and mental health.

### Empirical studies of chronic energy drink consumption and mental health outcomes

The literature search conducted for this review identified 17 articles that examined chronic energy drink usage in relation to mental health; three further articles were identified from reference lists. For details of all 20 studies, Table 2.

**Table 2.** Studies That Have Examined Associations Between Chronic Energy Drink Use and Mental Health Outcomes

*Study*	*Variables of interest*	*Design*	*Sample*	*Effects*
Arria *et al.*^56^	Depression (BDI)	Cross-sectional interviews and questionnaires (collected as part of a longitudinal study)	1097 fourth year US university students	No difference in BDI scores between frequent users and either infrequent users or nonusers
Azagba, Langille and Asbridge^57^	Depression (12-item version of the CES-D)	Cross-sectional survey (two-stage stratified cluster sample from three provinces)	8210 public school students (grades 7, 9, 10, and 12) in Canada	Higher depression associated with frequent (once a month or more) use
Evren and Evren^51^	Anxiety (PSTA) Depression (PSTA) Self-mutilation (unspecified) Suicidal thoughts (unspecified)	Cross-sectional online questionnaire	4957 10th grade students from 45 schools in 15 districts of Istanbul, Turkey (representative sample)	Frequency of energy drink use positively associated with anxiety. Frequency of energy drink use positively associated with depression. Frequency of energy drink use positively associated with self-harming behavior Frequency of energy drink use positively associated with suicidal thoughts Multivariate level: no association with anxiety or depression. Multivariate level: self-harming behavior and suicidal thoughts associated with consuming energy drinks every day
Hofmeister *et al.*^39^	Stress (DASS-21) Anxiety (DASS-21) Depression (DASS-21)	Cross-sectional online questionnaire	456 US veterinary students: University of Georgia (UOG; *N* = 227): Colorado State University (CSU; *N* = 229)	UOG: energy drink users had higher anxiety than nonusers (no differences for stress or depression); regular users had higher stress than nonregular users (no differences for anxiety or depression) CSU: energy drink users had higher anxiety than nonusers (no differences for stress or depression); regular users had higher depression, anxiety, and stress scores than nonregular users
Malinauskas *et al.*^53^	Jolt and crash episodes	Cross-sectional questionnaire	496 randomly surveyed US students	29% reported weekly jolt and crash episodes from energy drink use (significant dose-dependent effect)
	Heart palpitations			19% reported heart palpitations from energy drinks (marginally significant dose-dependent effect, *p* = 0.09)
Peters *et al.*^47^	PTSD symptoms after Hurricane Ike	Cross-sectional questionnaire	170 low-income at-risk African American/Latino male youth (9–19) from Houston, Texas	Initial associations between PTSD symptoms and 30-day prior use of antienergy drinks (significant) and energy drinks (marginally significant, *p* = 0.09)
				Multivariate: no associations between PTSD symptoms and energy drink or antienergy drink use
Pettit and DeBarr^44^	Stress (items from PSS)	Cross-sectional online questionnaire	136 US undergraduate students	Significant positive relationships between perceived stress and three measures of energy drink consumption
				Relationships between perceived stress and three other measures of energy drink consumption were not significant
Richards *et al.*^37^	General health (WPQ single-item)	Cross-sectional questionnaire	2030 British secondary school children	High consumption of caffeinated soft drinks/gum factor (comprising energy drinks, cola, and chewing gum) derived from the DABS was associated with low general health; remained significant after controlling for other dietary, demographic, and lifestyle factors
Richards and Smith^46^	Stress (WPQ single-item)	Cross-sectional questionnaire	2307 British secondary school children	Caffeine from energy drinks not associated with stress, anxiety, or depression at the univariate level
	Anxiety (WPQ single-item)			Marginally significant associations between low caffeine consumption (0.1–133 mg/w) from energy drinks and high stress and anxiety after controlling for additional dietary, demographic, and lifestyle factors; no effects for high ≥133 mg/w consumption
	Depression (WPQ single-item)			No association between caffeine from energy drinks and depression at the multivariate level
Ríos *et al.*^45^	Academic Stress (questionnaire Adapted from the Systemic Cognitive Model of Academic Stress)	Cross-sectional questionnaire (administered in August, participants asked to answer retrospectively for January–May). Representative stratified sample of medical-based subjects	275 first- and second-year Puerto Rican students	Energy drink consumption not associated with academic stress
				Soft drink and coffee consumption increased in times of high stress (although no effects regarding energy drinks, tea, and hot chocolate)
				49% reported that consuming caffeinated beverages was useful for coping with stress, with 42.6% admitting they would probably use caffeinated beverages as a stress coping strategy in the future
Rizvi *et al.*^43^	Increased consumption of caffeine/energy drinks (did not isolate energy drinks)	Cross-sectional questionnaire (although asked if participants had experienced increases/decreases in consumption in relation to pre-examination stress)	226 second-year medical students in Karachi, Pakistan	Increased consumption of coffee, tea, and energy drinks in 38.94% of respondents at pre-examination time
Snipes *et al.*^55^	Anxiety sensitivity (SURPS)	Cross-sectional online questionnaire	757 US undergraduate students	AmED users scored lower on anxiety sensitivity compared to alcohol-only users
	Hopelessness (SURPS)			No difference between AmED users and alcohol-only users for hopelessness
Stasio *et al.*^52^	Anxiety (BAI)	7-day retrospective survey (questionnaire)	107 young adults (college student athletes, Reserve Officers Training Corps cadets, and psychology students)	Energy drink use explained 29% of variance in anxiety scores (after controlling for sleep quality, coffee, tea, and soft drink consumption)
Toblin *et al.*^41^	Sleep disruption due to stress	Cross-sectional questionnaire (although design is not formally stated)	988 male US Army and Marine combat platoons deployed in Afghanistan in 2010 (initially 1249 surveyed using a cluster sample, 1000 consented to their data being used for research purposes, 988 answered energy drink question)	Those consuming ≥3/d more likely to report sleep disruption related to stress
				No differences between 0, 1–2, and ≥3/d on level of concern regarding not getting enough sleep
				Those consuming ≥3/d more likely to report sleep disruption on more than half the nights in the past 30 days because of stress related to combat, personal life, and illness
Trapp *et al.*^40^	Stress (DASS-21)	Cross-sectional questionnaire (population-based sample from the Western Australian Pregnancy Cohort (Raine) Study, a prospective cohort followed from gestation to early adulthood)	1062 young adult Australians	Univariate: energy drink consumption associated with depression (total sample, and males, but not females), anxiety (total sample, males, females), and stress (total sample, males, females)
	Anxiety (DASS-21)			Multivariate (most conservative model): only significant relationship was between energy drink use and anxiety in males
	Depression (DASS-21)			Multivariate: ≥250 mL/d energy drink users (compared to 0 mL/d) had higher anxiety and stress (total sample, and males, but not females), but not depression
				Multivariate: total sample: 100 mL/d energy drink consumption associated with anxiety and depression, but not stress
				Multivariate: males: 100 mL/d energy drink consumption associated with stress and anxiety, but not depression
				Multivariate: females: 100 mL/d energy drink consumption not associated with stress, anxiety, or depression
Vilija and Romualdas^49^	PTSD symptoms after lifetime traumatic experiences (IES-R)	Cross-sectional questionnaire (10 secondary schools randomly selected from 15 city districts in Kaunas, Lithuania)	1747 eighth grade pupils from Lithuania	PTSD symptoms associated with energy drink use (controlled for gender, index trauma, physical activity, smoking, and sense of coherence)
Waits *et al.*^42^	Change in energy drink use from predeployment to deployment in Operation Enduring Freedom	Cross-sectional questionnaire	183 deployed International Security Assistance Force personnel in Afghanistan	Increase in weekly consumption of Rip-It^®^ (significant) and Tiger^®^ (not significant) and decreases in Red Bull^®^, Monster^®^, and Rockstar^®^ (not significant)
				Overall change in total number of consumers of energy products from predeployment to deployment was not significant (although this also included other energy products, such as soda, coffee, Hydroxycut^®^), although number of servings per week increased from 16.6 (predeployment) to 24 (deployment)
Walther *et al.*^36^	Well-being (based on questions from the HBSC, KIGGS, and MDMQ)	Cross-sectional online questionnaire	500 adolescents and young adults (14–24 years old) from all provinces in Austria	Proportion with high well-being (55%) was higher in those who consumed energy drinks and alcohol once a week or less
				Proportion with low well-being was higher in those who consumed energy drinks and alcohol two to six times a week, daily, or several times daily
Wing *et al.*^38^	Mental health status (GHQ-12)	Cluster randomized controlled trial with 14 schools in Hong Kong	3713 (1545 intervention, 2168 control) secondary school (7th–11th grade: 12–18-year-old) students from Hong Kong	Lower incidence of consuming energy drinks in the intervention group
	Emotional problems (SDQ)			Improvement in GHQ-12 score in intervention group compared to control
	Conduct problems (SDQ)			Improvements in total difficulty, conduct, and hyperactivity in intervention group compared to control
	Peer relationships (SDQ)			No differences between groups for peer relationships, emotional problems, or prosocial behavior
	Hyperactivity/inattention (SDQ)			
	Prosocial behaviors (SDQ)			
Yudko and McNiece^54^	Depression (BDI II)	Prospective quasiexperimental	69 polydrug users (19 males, 50 females) receiving substance abuse treatment in a rural area of Hawaii	No association between having had an energy drink in the previous hour and BDI
	State anxiety (STAI)			No association between having had an energy drink in the previous hour and state anxiety
	Trait anxiety (STAI)			No association between having had an energy drink in the previous hour and trait anxiety

This table does not include case reports (Table 1) or studies that only investigated short-term effects (see Acute effects of energy drink consumption on mood section).

AmED, alcoholic energy drink; BDI, Beck Depression Inventory; CES-D, The Center for Epidemiologic Studies Depression Scale Revised; DABS, Diet and Behavior Scale; DASS-21, Depression Anxiety Stress Scale-21; GHQ-12, General Health Questionnaire-12; HBSC, health behavior in school-aged children; IES-R, impact of event scale-revised; KIGGS, Study on the Health of Children and Adolescents in Germany; MDMQ, Multidimensional Mood Questionnaire; PSS, Perceived Stress Scale; PSTA, Psychological Screening Test for Adolescents; PTSD, post traumatic stress disorder; SDQ, Strength and Difficulties Questionnaire; STAI, State-Trait Anxiety Inventory; SURPS, Substance Use Risk Profile Scale; WPQ, Well-being Process Questionnaire.

Walther *et al.*^36^ investigated associations between energy drink use and well-being in 500 adolescents and young adults from Austria. The study found a higher proportion of high well-being in those who consumed energy drinks and alcohol once a week or less, and a higher proportion of low well-being in those who consumed alcohol and energy drinks twice a week or more. An issue with this article was that the authors considered alcohol and energy drinks together, making it impossible to determine their individual effects. However, the results presented make it plausible to believe that frequent energy drink consumption may have been associated with low well-being. This was similar to the findings of Richards *et al.*^37^: high consumption of a factor analyzed variable named “Caffeinated Soft Drinks/Gum” (derived from the consumption of energy drinks, cola, and chewing gum) was associated with low general health in a large sample of British secondary school children (*N* = 2030), although the analysis presented did not isolate the effects of energy drinks themselves.

Wing *et al.*^38^ conducted an intervention study to improve sleep knowledge in a large sample (*N* = 3713) of secondary school children from Hong Kong. The article reported improvements in the intervention group relative to the control regarding sleep knowledge, mental health status, total difficulty, conduct problems, and hyperactivity, although no differences were observed for peer relationships, emotional problems, or prosocial behavior. What was of interest to the current review was that the intervention group was significantly less likely to consume energy drinks thrice a week or more compared to the control. Although it is not possible to tell from the data reported whether this observation was in any way associated with the changes in mental health, it is conceivable that it may have been. Furthermore, the study does provide hope that such interventions might be effective in reducing energy drink consumption and also in promoting better sleeping habits and mental health in adolescents. The next three sections will aim to address whether energy drink use is associated with stress, anxiety, and depression.

#### Stress

The studies identified that examined energy drink use in relation to stress generally reported positive relationships. Hofmeister *et al.*^39^ found higher stress levels in regular energy drink users (i.e., those who consumed more than one per week) compared to nonregular users (i.e., those who consumed one or fewer per week) in two samples of students. However, no differences were detected between energy drink users (i.e., those who consumed one or more per month) and nonusers. Trapp *et al.*^40^ measured energy drink consumption in mL/d, with estimates being based on frequency of consumption in days per month/week, and the amount of consumption in usual number of cans consumed on a day in which energy drinks were used. The study found stress to be positively associated with energy drink use in a large sample (*N* = 1062) of young adult Australians. Although these initial relationships disappeared once other factors were controlled for, higher stress did remain associated with consuming ≥100 mL/d or ≥250 mL/d. Compared to nonusers, consumption of ≥250 mL/d was associated with higher stress levels in the whole sample and in males separately. However, no such effect was observed in females. Furthermore, although males who consumed ≥100 mL/d reported higher stress levels than nonconsumers, no such effects were observed in females or in the sample as a whole.

In a large study (*N* = 988) of US Army and Marine personnel stationed in Afghanistan, Toblin *et al.*^41^ found that those who consumed three or more energy drinks per day were more likely to report sleep disruption due to stress. However, another study of International Security Assistance Force personnel in Afghanistan (Waits *et al.*^42^) found that, out of four energy drinks investigated, the number of servings consumed per week only increased significantly for one brand between predeployment and deployment. This effect was also considered likely to reflect the differential availability of brands between the United States and Afghanistan, and so may have been unrelated to the increased stress levels associated with military deployment. Furthermore, certain factors associated with military deployment make these data more difficult to interpret than studies that use nonmilitary samples. For instance, increased mental and physical requirements, as well as dysregulated sleep, might account for increases in both stress and energy drink use, and the two may not necessarily be causally linked.

Potential for stressful situations to be associated with increased use of energy drinks was provided by Rizvi *et al.*^43^ This study found that 38.94% of a Pakistani student sample claimed to have increased their coffee, tea, and energy drink consumption during pre-examination time. However, a limitation of the study was that it did not report the use of these products individually, making it impossible to relate the findings specifically to energy drinks. Furthermore, findings from this study should be interpreted with caution in light of the fact that the authors made a number of unsubstantiated claims. For instance, they stated, “it was observed that the intake of caffeine, tea and energy drinks most commonly affected metabolism, immunity, moods and sleeping patterns, which is in accordance with studies previously published” (p. 153), when their research was cross sectional in nature and no inferential statistics were reported. The authors also stated that “According to our study the students consume increased amounts of energy drinks and caffeine in the form of coffee, tea because they think it helps lift their mood and improves alertness” (p. 154), although no reasons for using the products were actually reported. The authors then concluded by saying “In light of the statistics obtained through this research, we recommend that students should inculcate physical activity and regular praying in their lives to combat stress effectively” (p. 154). This suggestion is again unfounded as no links between such activities and stress levels were reported in their article.

Pettit and DeBarr^44^ investigated whether perceived stress in undergraduate students was related to six measures of energy drink consumption. Significant positive correlations were observed with the following three measures: (1) number of days on which at least one energy drink was consumed in the previous 30 days, (2) average number of days per week on which energy drinks were consumed in the previous 30 days, and (3) the largest number of energy drinks consumed on any occasion in the previous 30 days. Although relationships with the number of energy drinks consumed the previous day, number of days on which energy drinks were consumed in the previous 7 days, and the approximate number of energy drinks consumed on days in which energy drinks were consumed in the previous 30 days were not significant, the effects were all in the same (positive) direction.

Ríos *et al.*^45^ found no difference between those who consumed energy drinks and those who did not regarding academic stress in a sample of Puerto Rican university students, even though nearly half of those surveyed claimed that using caffeinated products was useful for coping with stress. However, although soft drink and coffee consumption appeared to increase in times of high stress, no such effects were observed regarding energy drinks, hot chocolate, or tea. When interpreting these findings, it should be noted that the questionnaires were administered in August, and participants were asked to answer retrospectively from January to May (term time), potentially leading to recall bias.

Richards and Smith^46^ investigated associations between caffeine and stress, anxiety, and depression in a large cohort (*N* = 2307) of British secondary school children; the study also differentiated between caffeine consumed from energy drinks, cola, coffee, and tea. Though stress levels were positively related to total weekly caffeine intake (and remained so after other dietary, demographic, and lifestyle covariates had been controlled for statistically), no univariate association with caffeine from energy drinks was observed. At the multivariate level, however, low (0.1–133 mg/w) caffeine consumption from energy drinks was related to high stress levels, although no such observation was made regarding high consumption (≥133 mg/w), and the overall effect was only marginally significant (*p* = 0.067).

Two studies were identified that examined energy drink usage in relation to posttraumatic stress disorder (PTSD). Although it is acknowledged that this phenomenon should not be classified under the broader definitions of stress used by other studies discussed in this section, a consideration of their findings is still deemed to be useful. Peters *et al.*^47^ investigated substance use by Houstonian youth following Hurricane Ike. Actively trying to avoid thinking about the event was associated with a prior 30-day use of antienergy drinks (sometimes referred to as “relaxation drinks”; drinks that include ingredients such as melatonin, kava, valerian, and tryptophan, which are marketed with claims of promoting calmness and relaxation^48^) and energy drinks, although the latter effect was only marginally significant (*p* = 0.09). However, although the effect regarding antienergy drinks was retained in an unadjusted logistic regression model, which controlled for additional substance use, neither effect was significant when an adjusted model was used.

Vilija and Romualdas^49^ found PTSD symptoms to be positively correlated with frequency of energy drink consumption in a large sample (*N* = 1747) of Lithuanian secondary school children, even after controlling for sex, index trauma, physical activity, smoking, and sense of coherence. This article appeared to measure energy drink consumption based on a previously published food frequency questionnaire,^50^ which measured weekly intake using seven possible responses: “never,” “less than once a week,” “about once a week,” “two to four days a week,” “five to six days a week,” “once a day, every day,” and “every day, more than once.” It should, however, be noted that a number of other food products were also associated with PTSD symptoms (e.g., light alcoholic drinks, spirits, soft drinks, flavored milk, coffee, fast food, chips, salty snacks, processed foods). More importantly, energy drinks appeared to have been grouped together with sports drinks, which may have confounded the analysis.

#### Anxiety

Hofmeister *et al.*^39^ (described previously) found anxiety levels to be higher in energy drink consumers compared to nonconsumers in two samples of students. However, anxiety was only higher in regular users compared to nonregular users in one of the two samples, making it difficult to conclude whether such a relationship may be dose dependent or not. In addition to this, Evren and Evren^51^ reported energy drink use in a very large sample (*N* = 4957) of 10th grade students from Turkey to be associated with anxiety; compared to nonuse in the past year, anxiety scores were higher in those who had used the products once in their lifetime, once to three times in a month, once to five times a week, and every day. However, the effects did not remain significant at the multivariate level.

Stasio *et al.*^52^ found that 29% of anxiety scores in a sample of young adults comprising college student athletes, Reserve Officers Training Corps cadets, and psychology students were explained by energy drink consumption (measured in terms of the number of cans consumed in the previous 7 days); even one's sleep quality and other caffeinated drink consumption were controlled for statistically. In a similar manner, Trapp *et al.*^40^ (described earlier) observed anxiety scores to correlate positively with energy drink use in their total sample, as well as in males and females separately. However, in their most conservative multivariate analysis, the effect only remained significant in males. Those who consumed either ≥100 mL/d or ≥250 mL/d were also found to report higher anxiety levels than nonconsumers; these effects were observed in both the total sample and in males, although not in females. In addition to these findings, a study of US university students (Malinauskas *et al.*^53^) investigated energy drink use in relation to heart palpitations and “jolt and crash episodes.” The authors of the study defined this latter term as relating to “a feeling of increased alertness and energy (the jolt) followed by a sudden drop in energy (the crash).” The study observed a dose-dependent relationship between experiencing weekly jolt and crash episodes and the total number of energy drinks consumed at one time. A similar association was also reported for heart palpitations, although was only marginally significant (*p* = 0.09).

Richards and Smith^46^ (described earlier) observed that total weekly caffeine consumption was positively related to anxiety. When differentiating between sources of the substance, no univariate association between caffeine from energy drinks and anxiety level was observed. Although a trend (*p* = 0.087) was detected for low consumption (0.1–133 mg/w) of caffeine from energy drinks to be related to high anxiety at the multivariate level, no association was made with high consumption (≥133 mg/w), and the overall effect was not significant.

Although most studies have reported positive relationships, Yudko and McNiece^54^ found no association between trait or state anxiety and having used energy drinks in the previous hour in a sample of polydrug users attending a rehabilitation clinic in Hawaii. However, considering the relatively small sample size (*N* = 69), and that only nine participants had consumed an energy drink in the previous hour, it is likely that this study lacked the level of statistical power required to detect such effects. In further relation to the use of other substances, a study of 757 undergraduate students conducted by Snipes *et al.*^55^ found that anxiety sensitivity was lower in users of alcoholic energy drinks (i.e., drinks in which alcohol and energy drinks are mixed together) compared to alcohol-only users. The explanation given by the authors was that people with high anxiety sensitivity might avoid energy drinks due to the stimulant properties having potential to exacerbate their symptoms.

#### Depression

Although Richards and Smith^46^ (described earlier) reported positive associations between total weekly caffeine intake and depression scores in British secondary school children, no significant findings were made when caffeine intake from energy drinks was investigated separately. Arria *et al.*^56^ also found no differences in depression scores between high-frequency energy drink users (i.e., ≥52 during the past year), low-frequency energy drink users (i.e., 1–51 in the past year), and nonusers in a large sample (*N* = 1097) of fourth year US undergraduate students. In a similar manner, Hofmeister *et al.*^39^ (described earlier) observed no differences between energy drink users and nonusers in two samples of US veterinary students. However, in one of these samples, regular users were found to report significantly higher depression scores than nonregular users. A very large study of Canadian schoolchildren (*N* = 8210) conducted by Azagba *et al.*^57^ also observed higher depression scores to be associated with using energy drinks once per month or more.

Trapp *et al.*^40^ (described earlier) found initial positive relationships between energy drink consumption and depression in a sample of young adult Australians. However, although these relationships were observed in both the total sample and in males, they were not observed in females and did not remain significant once other factors had been controlled for statistically. Interestingly, although consumption of ≥250 mL/d was not associated with depression in the total sample, or in males or females separately, those who consumed ≥100 mL/d reported higher levels of depression than those who did not consume energy drinks at all. However, this relationship was observed only in the total sample and not in either sex independently.

Evren and Evren^51^ (described earlier) observed positive associations between energy drink use and depression, self-harming behavior, and suicidal thoughts in 10th grade students from Turkey. In each case, the effects appeared to be dose dependent. Although the relationships with depression disappeared at the multivariate level, self-harming behavior and suicidal thoughts remained associated with consuming energy drinks every day compared to not at all. Snipes *et al.*^55^ (described earlier) reported no difference in hopelessness scores between users of alcoholic energy drinks and alcohol-only consumers, and Yudko and McNiece^54^ (described earlier) observed no relationship between depression scores and having consumed an energy drink in the previous hour.

## Discussion

### Energy drink use and mental health

Although acute mood effects associated with energy drinks appear often to be positive, chronic use tends to be associated with undesirable mental health effects. Ten studies were identified that examined stress or stress-related outcomes in relation to energy drink use. Of these, two studies investigated PTSD: one reported a significant positive association,^49^ whereas the other did not.^47^ Three further studies did not include a direct measure of stress,^41–43^ although one of them (Toblin *et al.*^41^) did report a positive association between energy drink consumption and instances of sleep disruption due to stress. Of the five studies that did provide direct measurements of energy drink consumption and stress, two^45,46^ reported no association; the other three^39,40,44^ each reported positive relationships, as well as null findings, depending on which analyses were evaluated. For example, Hofmeister *et al.*^39^ presented findings from two different samples and also compared between energy drink users and nonusers, as well as between regular users and nonregular users. Some of these analyses yielded significant results, whereas others did not. Quantifying the overall outcome of such studies in relation to those that presented more straightforward analyses was therefore difficult. Similar issues relating to three studies^39,40,51^ were also encountered when discussing findings relating to anxiety and depression.

Eight studies investigated energy drinks and anxiety, or anxiety-related variables. Malinauskas *et al.*^53^ utilized indirect measures: a positive association was observed with weekly jolt and crash episodes, but the relationship with heart palpitations was not significant. Of the seven studies that provided direct measures, two^46,54^ reported no significant relationships, one^52^ reported a positive relationship, three^39,40,51^ reported both positive relationships and null findings, depending on which analyses were examined, and one^55^ reported a negative relationship. However, it should be noted that this last study compared consumers of alcoholic energy drinks to alcohol-only users, whereas the other studies listed investigated associations with energy drinks in the absence of alcohol.

Eight studies examined depression in relation to energy drink use. Snipes *et al.*^55^ investigated a related concept, “hopelessness,” although no association was found with alcoholic energy drink use. Of the seven studies that provided direct measures, three^46,54,56^ reported no significant relationships, one^57^ reported a positive relationship, and three^39,40,51^ reported both positive relationships and null findings. In addition, Evren and Evren^51^ also reported positive associations between energy drink use and self-harming behavior and suicidal thoughts.

From the studies identified that related to stress, anxiety, and depression, only one (Snipes *et al.*^55^) reported a negative association with energy drink use. Although null findings were also observed, a considerable number of studies reported positive relationships. This latter observation was therefore in line with the case reports identified in the area, which associated energy drink usage with a number of mental health conditions, as well as other studies that reported positive associations between energy drink use and low well-being^36^ and general health.^37^

### Potential mechanisms of action

As caffeine consumption itself has been associated with a number of psychiatric disorders,^9^ the findings reported in the current review uphold the idea that effects observed in relation to energy drinks may be dependent on caffeine.^7^ Although the lack of evidence for causality may be in accordance with the idea that caffeine use and mental health problems are simply correlated,^8^ the vast majority of studies identified here utilized cross-sectional designs. In most cases therefore, causality or direction of effect could not be inferred. One possibility is that those with low well-being, or mental health problems such as depression and anxiety, self-medicate^58^ by using energy drinks as a short-term “pick me up.” This usage pattern could therefore explain how the acute mood effects are often positive, whereas the long-term associations are not. Support for this idea is provided in that students are known to use caffeine as a coping strategy during stressful situations.^59,60^

Another possibility is that positive relationships observed between energy drink use and mental health problems are mediated by dysregulated sleep. However, determining the direction of such relationships may be difficult. Sleep debt could, for instance, cause fatigue leading to increased use of energy drinks. Conversely, as caffeine is known to interfere with sleep, sleep loss could lead to symptoms associated with mental health problems. It may also be that the relationship is bidirectional. For instance, children are known to use caffeinated products to remain awake at night when using media-related technology,^61^ and students have reported using energy drinks to combat the effects of insufficient sleep.^53^

### Limitations and directions for future research

A limitation of the current article is that the search criteria did not address additional aspects of mental health such as schizophrenia, personality disorder, and suicidality. Although some of the case reports identified suggest that energy drink use may be associated with such outcomes, it was deemed beyond the scope of the current article to examine them in greater detail. As this review aimed instead to focus more upon stress, anxiety, and depression, these phenomena may therefore be an area of interest for future research.

The majority of studies identified were conducted in young populations, potentially reflecting their comparatively high consumption of energy drinks. However, the disproportionately large number of studies utilizing university students might be due to the relative ease in which such participants can be recruited. Studies into younger populations may therefore be meritorious because children are both targeted by energy drinks advertisers and likely to be relatively naive caffeine consumers. In addition, research into older populations may be of interest. For instance, late adolescence and early adulthood are associated with the onset of psychiatric disorders and stress associated with adjustment to many life changes, and also represent populations that are likely to report energy drink use. For these reasons, the question should be asked as to whether the relationships observed are unique to young persons or are also found in older populations.

As the majority of studies identified in this review were cross sectional, longitudinal and intervention studies are required. An example of a study design that may be useful for investigating causality and direction of the relationships in question comes from Wing *et al.*^38^ who conducted an intervention involving sleep education. Those in the intervention condition improved in terms of mental health and, additionally, reduced their energy drink intake. Further studies of this nature could avoid ethical concerns regarding administering energy drink products to participants, while furthering our knowledge of how sleep, energy drink use, and mental health outcomes are related. Longitudinal studies could also be used to track changes in energy drink consumption and mental health status over time. By doing this, analyses could be conducted to investigate whether changes in consumption are predictive of changes in mental health outcomes.

## Conclusions

The current article has aimed to provide a review of the literature relating to energy drink use and mental health. Because most of the studies identified examined stress, anxiety, and depression, particular focus was placed on these areas. Although a number of studies investigating acute effects of energy drinks on mood reported benefits, only one such observation was made in relation to chronic use. Although null findings were also relatively common, most studies of chronic use provided evidence to suggest that energy drinks are associated with mental health problems. However, as almost all studies identified were cross sectional, and some did not control for other relevant factors, such as sex, socioeconomic status, and additional caffeine intake, the nature of these relationships is not yet fully understood. Therefore, to improve our understanding of such phenomena, longitudinal and intervention studies are required.
